# Demographic Data, Clinical Characteristics, and Outcomes of Pediatric Patients Who Received Palliative Care in King Abdullah Specialized Children’s Hospital, Riyadh, Kingdom of Saudi Arabia

**DOI:** 10.7759/cureus.49032

**Published:** 2023-11-18

**Authors:** Wesam Althaqafi, Bader M Alqahtani, Mohammed A Khan, Ahmed A AlAbdulkarim, Abdullah Z Alkhars

**Affiliations:** 1 Pediatrics, King Abdullah Specialized Children's Hospital, Riyadh, SAU; 2 Pediatrics, Maternity and Children Hospital, Al-Ahsa, SAU

**Keywords:** symptom management, pain management, chronic pain, pediatric palliative care, palliative care, pediatric

## Abstract

Background

Palliative care is defined as a comprehensive care approach that improves the quality of life of patients and their families facing the problems associated with life-threatening illnesses by alleviating the pain by different means. The death of children receiving palliative care is mainly due to congenital anomalies (26% of infants) and cancer (17% of children). This study aims to identify the demographic data, clinical characteristics, and outcomes of patients who received specialized pediatric palliative care (PPC) services in a tertiary care center in Saudi Arabia.

Method

This five-year retrospective chart review examines all children who received specialized palliative care services at King Abdullah Specialized Children’s Hospital (KASCH), Riyadh, Saudi Arabia, from 2016 to 2021. The data include the sociodemographic and clinical characteristics, as well as the referral process information and the used PPC interventions.

Results

A total of 138 patients were included in this study. The gender distribution was 50.40% male and 49.60% female. Children aged 1-10 years accounted for 52.9% (n=73) of the sample size. Malignancy was the predominant diagnosis. Tube feeding was the most common intervention provided (28%, n=39). The most common symptom was chronic pain (61.6%, n=85). Morphine was used in more than half of the patients (53%, n=73).

Conclusion

In our study, children between 1 and 10 years of age comprised 52.9% (n=73) of the total sample. Malignancy was the most common diagnosis. The most common reason for consulting the PPC unit was symptomatic treatment (87.7%, n=121). The symptom reported most commonly by children was chronic pain (61.6%, n=85). The medical technology most commonly used was tube feeding (28%, n=39). The most common medication given to patients was morphine (53%, n=73). To sum up, identifying the demographics and clinical characteristics of children who previously required PPC would help healthcare professionals identify future cases in need of PPC.

## Introduction

Palliative care for children starts at diagnosis and encompasses children with a set of life-limiting and life-threatening conditions. Pediatric palliative care (PPC) is designed to help children and their families deal with their medical conditions while enabling them to live life to the fullest. As per the World Health Organization (WHO), health-related quality of life is a multi-dimensional concept, including physical, social, mental, and spiritual aspects [1]. The WHO defines palliative care as a comprehensive care approach that improves the quality of life of patients and their families facing problems associated with life-threatening illnesses, through the prevention and relief of suffering by means of early identification and impeccable assessment and treatment of pain and other physical, psychosocial and spiritual problems [2]. The WHO definition of PPC is more a philosophical, rather than a dictionary concept, as it does not just report what palliative literally means (the alleviation of suffering), but elicits certain values (e.g., patient-centeredness, holism, and multidisciplinary) that guide action and improve practice. The primary aim of palliative care is to preserve the quality and the meaningfulness of life for both patients and their families. Unlike hospice care, palliative care is not limited to terminal care but is appropriate for patients in all disease stages [3]. In 2014, the WHO recognized palliative care as a fundamental part of comprehensive care throughout the life course. Since the innovation of advanced medical technologies, palliative care has been bringing aspiration in the alleviation of suffering and distress [4].

Conventionally, palliative care was restricted to cancer patients. Nowadays, however, the scope of palliative care has been expanded to also include noncancer patients. PPC services provide care for children and young adults with a wide range of life-limiting conditions, including neurological, genetic, respiratory, and metabolic conditions, as well as malignancy. In 2014, Virdun et al. in their systematic review found that the deaths of pediatric patients receiving palliative care were mostly due to congenital anomalies (26% of infants), cancer (17% of children), and CNS diseases (11% of children) [5]. In terms of survival rate, unlike adults, pediatric patients receiving palliative care remain alive for more than one year after launching the PPC program [6]. In 2004, a population-based survey in the United Kingdom showed that 16.8% of people who died from nonmalignant conditions had at least the same level of need for palliative care as patients with malignant conditions [7].

Early integration of palliative care into the treatment plan of patients with limiting conditions results in less suffering, more enhanced well-being, and better outcomes [2]. It has been noticed recently that the number of children’s hospitals that provide specialized palliative care services has been mounting, to meet the demands of children with limiting conditions [6,8]. The efficacy of PPC is based on a multidisciplinary approach. The team of PPC consists of physicians, nurses, and allied healthcare professionals, such as social workers, occupational therapists, and physical therapists, all of whom work together to provide care through a consultative model [6]. The psychological assessment and intervention of patients are crucial to leverage the quality of care in PPC. As for somatic pain, in patients with limiting conditions, ongoing tissue damage is the main source of pain, which might be caused by the pathologic process of the respective condition, recurrent harms, and injuries and invasive procedures either for diagnostic or therapeutic purposes. The estimated percentage of pediatric patients with life-limiting conditions who suffer from drastic pain with significant impact is approximately 3%-5% [9,10].

The oral route is the favorable choice for the administration of pain management medications as it is the easiest for patients, has high efficacy, and is the least painful. The WHO guidelines for pain management in children and young people with medical conditions include non-opioid analgesics, opioids, local anesthetics, and adjuvant medications [9].

When taking care of children with life-limiting conditions, it is imperative to enrich and upskill the family and the medical team with appropriate equipment, such as beds, water or air mattresses, domiciliary oxygen, suction, infusion, and feeding pumps. Moreover, the availability of a 24-hour hotline, easy access to nearby health care centers, psychosocial support, sibling assistance, and weekly support groups for grieving families might be applied and could provide great influence. Finally, providing different settings of care such as ambulances, emergency rooms, inpatient beds, and home health care has been associated with excellent outcomes [5,11].

PPC in the Middle East and North Africa is still lagging behind the developed countries due to some drawbacks, such as lack of advanced knowledge, insufficient financial support, and lack of policymakers. Nevertheless, lately, PPC in the Middle East has been evolving through strategies such as training human resources, adding PPC rotations into the residency curriculum, and supporting research projects in this field [12]. Even though the field of PPC is growing, there is an existing gap in our region in terms of demographic data, clinical characteristics, and outcomes for pediatric patients in need of palliative care [6].

To the best of our knowledge, we are the first in Saudi Arabia to study PPC in the context of demographic data, clinical characteristics, and patients’ outcomes.

This study aims to identify demographic data, clinical characteristics, and outcomes of pediatric patients who received palliative care in King Abdullah Specialized Children’s Hospital, Riyadh, Kingdom of Saudi Arabia, from 2016 to 2021.

## Materials and methods

Study design and settings

This is a descriptive retrospective study that included a total of 138 pediatric patients who were referred to King Abdullah Specialized Children’s Hospital (KASCH), a center under the governance of the Ministry of National Guard and Health Affairs, for palliative care services from 2016 to 2021.

Study subject

All pediatric patients who had been referred for PPC from January 2016 to December 2021 were included in this study.

Data collection

All the data in this study was retrieved from the BestCare system, which is the database of patients used at King Abdullah Specialized Children’s Hospital. A standardized data collection sheet was generated to enter the data, and the data were then de-identified. The collected data included demographic data (age, gender, nationality, and date of death), clinical characteristics (main diagnosis, reason for referral, code status, use of medical technology: tube feeding, oxygen support, central line, dialysis, mechanical ventilation, tracheostomy, drain, urinary catheter, ventriculoperitoneal (VP) shunt, and social information (health insurance coverage). The interventions recommended by the team of PPC were extracted from their progress notes and orders and included symptom management, moral support, prescription or recommendation of pharmacological interventions, and/or medical technology.

Definitions

Medical technology was defined as any used machine that helps and improves the patient’s status, including tube feeding, O2 support, central line, VP shunt, tracheostomy, clean intermittent catheterization/Foley catheter, drain, dialysis, and mechanical ventilation. Insurance refers to the eligibility status of patients. The code status decision was represented as either full code, supportive, or comfort.

Data management and statistical analysis

Data analysis was performed using Statistical Product and Service Solutions (SPSS, version 23) (IBM SPSS Statistics for Windows, Armonk, NY). Descriptive statistics and graphic analysis were used to analyze the data. Categorical variables were reported as frequency and percentage values. Continuous variables were reported as mean and standard deviation. Results with a P-value < 0.05 were considered significant. A confidence interval of 95% was estimated for the prevalence.

Confidentiality and ethical consideration

All data were managed with total confidentiality. Privacy was ensured throughout the study steps. Ethical approval was obtained from the ethical board of King Abdullah International Medical Research Center, Riyadh, Kingdom of Saudi Arabia.

## Results

A total of 138 children were referred for palliative care services from several departments for the treatment of various conditions.

Demographics and characteristics of the patients

Table 1 shows the demographics and characteristics of the patients. Children between one and 10 years of age comprised 52.9% (n=73) of the total sample. The remaining 47.1% (n=65) was distributed between newborns, patients less than one year of age, and patients more than 10 years of age. The sex distribution of the study population was 50.7% (n=70) male and 49.3% (n=68) female. The major city from where most patients were referred was Riyadh (73.90%, n=102), followed by Jeddah (4.30%, n=6), Al-Qassim (2.90%, n=4), and AlAhsa (1.4%, n=2). Most patients were Saudi with a percentage of 92.80% (n=128). As for the code status of patients, 34.80% (n=48) had full code, 45.70% (n=63) had supportive care, and 19.60% (n=27) had comfort care. As high as 64.50% (n=89) of the patients who were referred for PPC passed away before or after being seen by the healthcare providers. As for the health insurance coverage, 75.4% (n=104) of the participants were eligible. The patients were classified into different categories based on the system the medical condition of each patient affects at the time of referral to the PPC team. It was observed that malignancy was the most common category (32.6%, n=45), followed by neurologic conditions (28.3%, n=39), genetic and metabolic conditions (16.7%, n=23), hematologic conditions (6.5%, n=9), cardiac conditions (5.8%, n=8), respiratory conditions (4.3%, n=6), and renal conditions (2.2%, n=3). As for the source of referral, 96.30% (n=133) of the patients were referred from inpatient settings, while the remaining 3.70% (n=5) were referred from the outpatient setting. Regarding the location of patients, 94.90% (n=131) of the patients were seen in the general ward. Concerning moral support, about 54.3% (n=75) received moral support.

**Table 1:** Demographics and characteristics of the patients (n=138)

Characteristics	Level	n	%
Age	< 1 Month	3	2.20
	1-12 Months	14	10.10
	1-5 Years	40	29.00
	6-10 Years	33	23.90
	11-15 Years	48	34.80
Gender	Male	70	50.40
	Female	68	49.60
City	Riyadh	102	73.90
	Jeddah	6	4.30
	Dammam	0	0.00
	Al Ahssa	2	1.40
	Al Madinah	1	0.70
	Qassim	4	2.90
	Others	23	16.70
Nationality	Saudi	128	92.80
	Non-Saudi	10	7.20
Code status	Full Code	48	34.80
	Supportive	63	45.70
	Comfort	27	19.60
Life status	Alive	49	35.50
	Dead	89	64.50
Insurance	Covered	104	75.40
	Not covered	34	24.60
Diagnosis	Cancer	45	32.60
	Non-Cancer	93	67.40
Source of referral	Inpatient	133	96.30
	Outpatient	5	3.70
Medical technology	Yes	61	44.40
	No	77	55.60
Location of patients	General Ward	131	94.90
	PICU	5	3.70
	Outpatient	2	1.50
Moral support	Yes	75	54.3
	No	63	44.2

Comparing cancer to non-cancer patients in terms of age group, gender, code status, life status, and insurance

Table 2 compares cancer to non-cancer patients in terms of age group, gender, code status, life status, and insurance. As for the age group, 82.5% (n=47) of children younger than six years of age were under the group of non-cancer patients. As for the code status, 79.2% (n=38) of the non-cancer group were signed as a full code with a p-value < 0.02. As for the life status difference between cancer and non-cancer groups, only 12.2% (n=6) of the cancer group were alive, while as high as 87.8% (n=43) of the non-cancer group were alive.

**Table 2:** Oncology and non-oncology patients’ characteristics (n=138) P-values were calculated using the chi-square test. * Significant p-value (<0.05).

Characteristics	Level	Cancer		Non-cancer		P-value
		n	%	n	%	
Age Group	< 6 years	10	17.5	47	82.5	0.002*
	≥ 6 years	35	43.2	46	56.8	
Gender	Male	21	30.4	48	69.6	0.55
	Female	24	35.3	44	64.7	
Code Status	Full Code	10	20.8	38	79.2	0.02*
	Supportive	28	44.4	35	55.6	
	Comfort	7	25.9	20	74.1	
Life Status	Alive	6	12.2	43	87.8	<0.001*
	Dead	39	43.8	50	56.2	
Insurance	Covered	35	33.7	69	66.3	0.65
	Not covered	10	29.4	24	70.6	

List of the most common reasons for consultation, patients’ symptoms, and provided interventions among cancer and non-cancer groups

Table 3 lists the most common reasons for consultation, symptoms, and provided interventions among cancer and non-cancer patients. The most common reason for consulting the pediatric palliative care unit was symptomatic treatment (87.7%, n=121), followed by moral support (31.9%, n=44). Other reasons for consultation account for less than 8.7% (n=12). It is worth mentioning that some patients had more than one symptom and reason for consultation. The symptom reported most commonly by patients was chronic pain (61.6%, n=85), followed by excessive secretions (32.6%, n=45), dystonia/autonomic dysfunction (29.7%, n=41), spasticity (20.3%, n=28), feeding intolerance (15.9%, n=22), and lastly constipation (12.3%, n=17). The cancer groups reported chronic pain as the most common symptom (40%, n=34), while the non-cancer group reported spasticity as the most common symptom (92.9%, n=26). As for the provided intervention, symptomatic treatment was provided for 32.2% (n=39) of the cancer group and for 67.8% (n=82) of the non-cancer group. Moral support was provided for 36.4% (n=26) and 63.6% (n=49) of cancer and non-cancer groups, respectively. Morphine was given to 48.6% (n=35) of the cancer group and 51.4% (n=37) of the non-cancer group. Diazepam was given only to 11.5% (n=3) of the cancer group, while up to 88.5% (n=23) of the non-cancer group. Medical technology was used in 21.7% (n=13) of the cancer group and in 78.3% (n=47) of the non-cancer group with a p-value <0.01.

**Table 3:** Reasons for consultation, patients’ symptoms, and provided interventions among cancer and non-cancer groups (n=138) P-values were calculated using the chi-square test. * Significant p-value (<0.05).

Characteristics	Level	Cancer		Non-cancer		P-value
		n	%	n	%	
Reason for Consultation						
Symptomatic treatment	No	6	35.3	11	64.7	0.06
	Yes	39	32.2	82	67.8	
Moral support	No	29	30.9	65	69.1	0.052
	Yes	16	36.4	28	63.6	
Patient’s Symptoms						
Chronic pain	No	11	20.8	42	79.2	0.019*
	Yes	34	40	51	60	
Excessive secretions	No	33	35.5	60	64.5	0.3
	Yes	12	26.7	33	73.3	
Dystonia/autonomic dysfunction	No	38	39.2	59	60.8	0.011*
	Yes	7	17.1	34	82.9	
Spasticity	No	43	39.1	67	60.9	<0.001*
	Yes	2	7.1	26	92.9	
Feeding intolerance	No	39	33.6	77	66.4	0.56
	Yes	6	27.3	16	72.7	
Constipation	No	40	33.1	81	66.9	0.76
	Yes	5	29.4	12	70.6	
Provided Interventions						
Symptomatic treatment	No	6	35.3	11	64.7	0.06
	Yes	39	32.2	82	67.8	
Morphine	No	10	15.2	56	84.8	<0.001*
	Yes	35	48.6	37	51.4	
Gabapentin	No	36	37.5	60	62.5	0.062
	Yes	9	21.4	33	78.6	
Diazepam	No	42	37.5	70	62.5	0.011*
	Yes	3	11.5	23	88.5	
Movicol	No	35	29.4	84	70.6	0.045*
	Yes	10	52.6	9	47.4	
Medical technology	No	32	42.7	43	57.3	0.01*
	Yes	13	21.7	47	78.3	
Moral support	No	19	31.1	42	68.9	0.66
	Yes	26	34.7	49	65.3	

Pediatric Palliative care interventions and management

**Figure 1:**
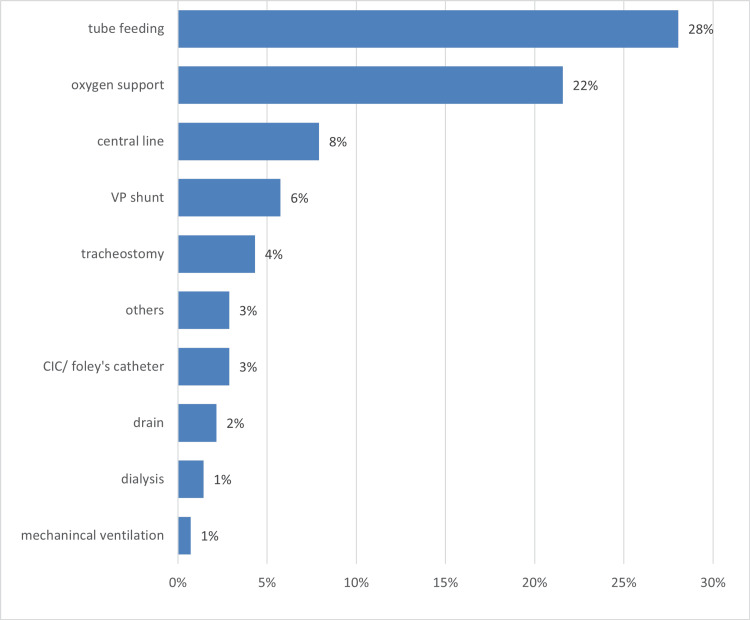
Medical technology provided for patients receiving PPC PPC, pediatric palliative care

Figure 1 represents the medical technologies provided for patients. The medical technology most commonly used was tube feeding (28%, n=39), followed by oxygen support (22%, n=30), central line (8%, n=11), VP shunt (6%, n=8), and tracheostomy (4%, n=6). It is worth mentioning that a few children were given more than one medical technology.

**Figure 2:**
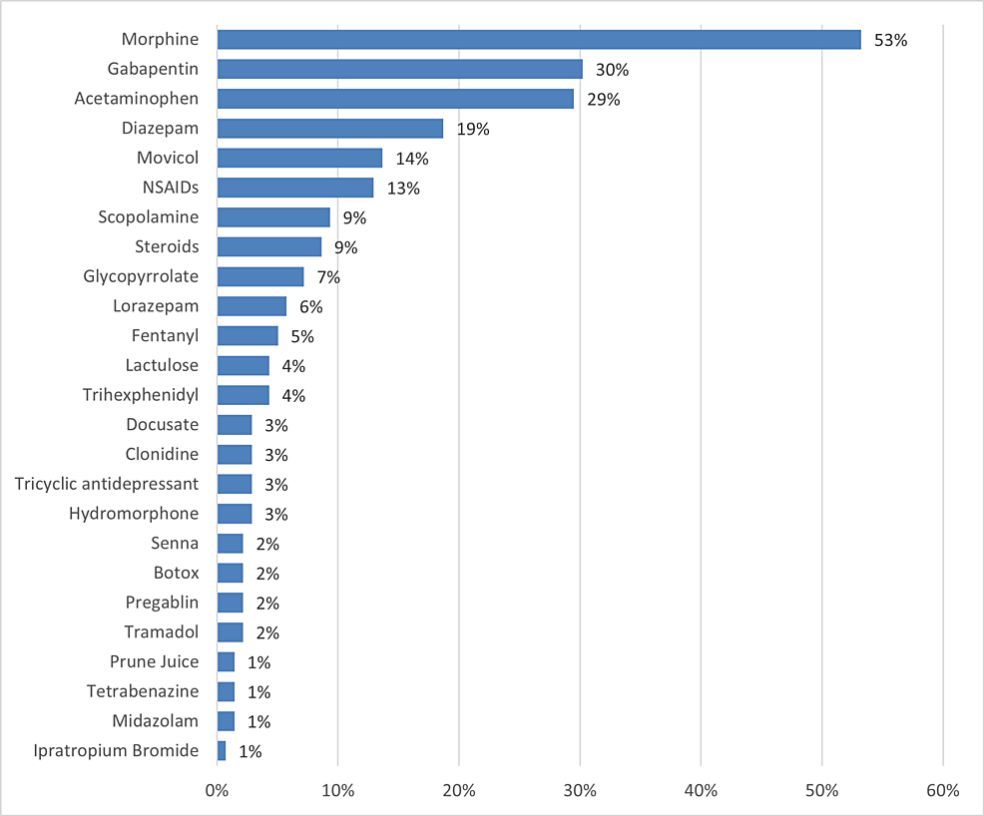
Medications given to patients receiving PPC PPC, pediatric palliative care

Figure 2 represents the medications given to patients. About 74% (n=102) of the children received medications. The most common medication used was morphine (53%, n=73), followed by gabapentin (30%, n=41) and acetaminophen (29%, n=40). The remaining medications, such as diazepam, movicol, and NSAIDs, were used for less than 20% of the patients. It is worth mentioning that a few children were given more than one medications.

## Discussion

Over the past few years, PPC in Saudi Arabia has gained increasing attention and recognition from the public, the medical sector, and the social services department.

In this paper, malignancy was the most common diagnosis among our sample (32.6%, n= 45), followed by neurologic conditions (28.3%, n= 39). These findings are consistent with the study of Feudtner et al. who found hemato-oncologic conditions (22%) and central nervous system conditions (19%) to be the most common diagnoses among children receiving PPC in the United States and Canada [6]. Our results are also consistent with the study of Rosenwax et al. who found neoplasms (59.5%) to be the most common diagnosis among Western Australians receiving PPC [7]. Presumably, the metastatic nature of cancers makes the disease relatively more disabling than the other conditions, which in turn necessitates the use of palliative care more frequently.

We found inpatient referrals for PPC to be far more common than outpatient referrals (96.3% vs. 3.7%). This is supposedly because most life-limiting conditions have an insidious progressive course, during which children’s health slowly deteriorates until reaching a stage where preserving lives or relieving symptoms cannot be achieved without the use of PPC. In such a case, rather than being outpatient, children are expected to get hospitalized frequently either in the general ward or in the PICU before eventually receiving PPC. Anyhow, given the chronically disabling nature of the life-limiting conditions necessitating palliative care for children, it is sensible to assume that the provision of home care as a standard service of PPC will take PPC a step forward and greatly expand the scope of care it provides, maximizing the quality of life for both children and their families. This is especially considerable when taking into consideration the fact that Chan in his study stated that home care is one of the most desirable models of palliative care [4], highlighting the need for enabling such flexible care, which includes psychosocial support, 24-hour professional care, respite care, and sibling support. In the previous literature, the easy accessibility to the services of PCC, reflected in the form of delivery of cross-site service (inpatient and outpatient care) and proximity of PPC services to the residence site, was considered as a central factor for achieving optimal PPC [6,8,10]. Therefore, providing this kind of care regardless of where children and their families live is considered one of the biggest challenges for PPC.

In our study, pain was the most common symptom, reported by 61.6% (n=85) of patients, followed by excessive secretions (32.6%, n=45) and dystonia/autonomic dysfunction (29.7%, n=41). This order of symptoms corresponds with the order that Irola Moya et al. found in their study despite the differences in symptoms’ percentages [11]. Furthermore, given that, in the study of Weaver et al., pain has been described as a terminal symptom of childhood cancer [2], it is no wonder that we found pain to be the most common symptom and morphine to be the most prescribed drug as cancer was the most common condition among our patients. As for excessive secretion and the subsequent breathlessness it results in, breathlessness support services (BSS) are an option to consider. BSS is a multi-professional integrated outpatient and home-care service that combines respiratory therapy, physical therapy, occupational therapy, and palliative care. Chan in his study found that among patients receiving palliative care, the treatment group whom BSS was used for had an improved survival rate at six months in comparison to the control group whom BSS was not used for [4].

Weaver et al. in their study stated that numerous international oncology groups support the early use of PPC for children with cancers as it has been associated with better symptom control and quality of life for both children and their families [2]. In our study, the majority of the oncology group (86.66%, n=39) and non-oncology group (88.17%, n=82) had their symptoms relieved by the early implementation of PPC services, reflecting the importance of palliative care, and supporting the statement mentioned in Weaver et al.'s study.

In this study, we found tube feeding to be the most common medical technology used for children receiving PPC (28%, n=39). Likewise, Feudtner et al. in their study found gastrostomy tubes to be the most common medical technology used for children receiving PPC (48.5%) [6]. Given the fact that oncologic conditions and CNS conditions were found to be the most common diagnosis in both our study and the study of Feudtner et al., we assumingly attribute the commonness of tube feeding used to treat nausea and anorexia caused by the tumors themselves, chemotherapy, and radiation in patients with oncologic conditions, as well as to dysphagia, hypotonia, and hyporeflexia in patients with CNS conditions, all of which can result in poor feeding and malnutrition, eventually necessitating the use of feeding tubes.

In King Abdullah Specialized Children's Hospital, the number of referred cases for PPC has increased from 44 cases in 2016-2017 to 49 cases in 2020-2021. This rise in the number of referred cases may simply reflect increased awareness, focus, and a cultural shift in how families and healthcare professionals view PPC services. We think that referring cases for the PPC through consultation services will continue to rise due to the continuous improvement and recognition of the PPC specialty. This emphasizes the necessity of establishing a well-structured PPC unit with appropriate staff, including physicians, nurses, members of social services, psychologists, and psychiatrists in each children's hospital, integrating palliative care into the rotations of pediatric residency programs, establishing guidelines in prescribing opioids, conducting research in palliative care, and finally engaging of nongovernmental organizations in the development of this kind of care.

Strengths

Identifying the demographics and clinical characteristics of children who require PPC would help healthcare professionals identify future cases requiring PPC. Although the American Academy of Pediatrics has previously highlighted the need for basic skills in palliative care, only 39% of healthcare professionals were found to have the necessary knowledge (7). Our research represents the largest data on PPC cases from Saudi Arabia to date, describing the demographics, clinical characteristics, and the provided interventions.

Limitations

Our report has its own limitations that can be addressed in future research. This study only includes data from a single pediatric center that is part of a military tertiary care facility in a large metropolitan city. Therefore, the results of this paper should not be generalized.

Recommendations

To fully comprehend the importance of PPC in Saudi Arabia, nationwide initiatives are required. Large-scale surveillance studies can be used to assess the knowledge and perceptions of PPC among pediatricians. To boost the knowledge of PPC among healthcare professionals, holding conferences and in-hospital activities concerning PCC can be attempted.

## Conclusions

In our study, children between two and 10 years of age comprised 58.1% of the total sample. Malignancy was the most common diagnosis, followed by neurologic conditions (28.3%). The most common reason for consulting the pediatric palliative care unit was symptomatic treatment (87.7%). The symptom reported most commonly by patients was chronic pain (61.6%). The medical technology most commonly used was tube feeding (28%). The most common medication given to patients was morphine (53%). Our research enriches the literature with the largest data on PPC cases from Saudi Arabia to date, describing detailed demographics, clinical characteristics, reasons for consultation, and clinical outcomes to help healthcare professionals notice and identify cases requiring PPC.
